# Clinical Subtypes and vHIT Parameters in a Population With Bilateral Vestibulopathy

**DOI:** 10.3389/fneur.2021.673974

**Published:** 2021-06-07

**Authors:** Fiorella Mancino-Moreira, Almudena Rueda, Jonathan Esteban-Sanchez, Eduardo Martin-Sanz

**Affiliations:** ^1^Department of Otolaryngology, University Hospital of Getafe, Madrid, Spain; ^2^Department of Neurology, Getafe University Hospital, Madrid, Spain; ^3^Department of Medicine, School of Biomedical Sciences and Health, Universidad Europea de Madrid, Madrid, Spain

**Keywords:** bilateral vestibular hypofunction, bilateral vestibulopathy, vHIT, head impulse test, subtypes

## Abstract

**Objective:** To evaluate the different peripheral, neurological, genetic, and systemic etiologies of bilateral vestibulopathy (BVP) and the value of vHIT in the diagnostic process.

**Materials and methods:** A retrospective case review was performed on 176 patients diagnosed with BVP in a tertiary referral center, between 2010 and 2020. Inclusion criteria comprised imbalance and/or oscillopsia during locomotion and horizontal angular VOR gain on both sides <0.8. We classified patients into different groups according to (1) their fulfillment of the Barany guideline for bilateral vestibulopathy (2) the definite etiology of BVP and (3) the four clinical subtypes distributed in our population (recurrent vertigo with BVP, rapidly progressive BVP, slowly progressive BVP, and slowly progressive BVP with ataxia). Medical history of vertigo, hypoacusis or migraine, and family background of imbalance and/or oscillopsia were assessed. Horizontal, posterior, and superior semicircular canal angular VOR gain was registered along with saccadic parameters such as velocity, and dispersion of the saccades' latency values.

**Results:** Barany's Society diagnostic criteria for BVP was accomplished in 89 patients. Among our patients, 13.6% had migraines in their medical history and the idiopathic group accounted for 50% of the population. All four clinical subtypes were found in our population, slowly progressive bilateral vestibulopathy without vertigo was the most frequent one. A percentage of our population could not be categorized into any of the former subtypes, many of these patients were diagnosed with BVP after suffering a single vertigo episode. Lower vHIT gains were found in those patients with Barany's criteria for BVP and oscillopsia was significantly more prevalent in this group.

**Conclusions:** Bilateral vestibulopathy manifests with very different patterns representing a very heterogeneous condition. The distribution of the clinical subtypes and Barany's criteria are a useful clinical tool to differentiate groups of patients. The vHIT can serve as an initial tool for identifying patients with BVP. The finding of bilateral vestibular involvement in a clinically suspected unilateral vestibulopathy should be considered in some patients.

## Introduction

Bilateral vestibulopathy (BVP) is a heterogeneous clinical condition characterized by a hypofunction of the vestibular nerves or labyrinths on both sides (1). Partially reduced or absent function of the vestibular organs and/or vestibular nerves results in different levels of impairment or total loss of the major vestibular functions: posture and balance control, gaze stabilization, and spatial orientation (2).

Spatial disorientation, oscillopsia, diminished dynamic visual acuity (DVA), and balance problems are the main deficits reported by patients with BVP, particularly in darkness and on uneven ground.

BVP is a disorder with different clinical pictures (combined or isolated deficits of the otolith and semicircular canal functions), it remains a diagnostic challenge, and therefore, its often under or misdiagnosed.

The most frequent etiologies of BVP include ototoxicity, Meniere's disease, infectious diseases, and genetic disorders (3). However, the etiology remains undetermined in 20–51% of the patients (4). Its estimated prevalence in adults is 28/100,000, and it accounts for 4–7% of dizziness/vertigo (5).

Four different clinical subtypes have been described as follows (3): (I) recurrent vertigo and BVP (II), rapidly progressive BVP (III), slowly progressive BVP, and (IV) BVP with neurological deficits. In a recent study (3) most non-idiopathic BVP patients presented a clinical subtype that would be expected due to its etiology.

Nevertheless, so far we know, there is no correlation between the clinical subtypes and vestibular test findings, but it is believed that stratifying the patient population may facilitate the identification of underlying causes for which there may be a genetic predisposition (6).

Many challenges are met when establishing the diagnosis of BVP. Currently, many different diagnostic tests are used for vestibular evaluation, such as the caloric test, rotatory chair tests, video head impulse test (vHIT), vestibular-evoked myogenic potentials (VEMP), DVA test (7).

In 2017, the Committee for the International Classification of Vestibular Disorders (ICVD) of the Barany Society proposed the diagnostic criteria of BVP (8). However, some controversy remains, and at this moment, no diagnostic standards regarding some clinical and vestibular test findings are available.

The vHIT is a test of the angular vestibulo-ocular reflex (VOR). A bilaterally reduced or absent angular VOR function has been included in the diagnostic criteria for BVP in the consensus document of the Classification Committee of the Bárány Society (8).

Recently Batuecas-Caletrio et al. (9) described that BVP patients with scattered saccades were more prone to oscillopsia independent of their gain values, suggesting that the degree of synchronization of the saccades in successive head impulses can be considered a useful measurement of compensation.

We aim to review our series of BVP patients, analyze their etiologies, identify and characterize the clinical subtypes, and correlate with audio-vestibular function and familiar aggregation.

## Materials and Methods

This is a retrospective study. Patients with a suspicion of BVP were included between January 1, 2010 and June 30, 2020.

Inclusion criteria were (1) a vHIT response from the six semicircular canals, below normal established limits for age (10, 11) and/or (2) the presence of unsteadiness when walking or oscillopsia during quick head movements and/or (3) worsening of unsteadiness in darkness and/or on uneven ground.

Clinical and demographic variables such as age, sex, personal and familial history, presence of imbalance, and/or oscillopsia were recorded.

The patients were then categorized depending on the fulfillment of the diagnostic criteria for BVP proposed by the Barany Society (8).

The patients were also classified in the four clinical subtypes previously described: (I) recurrent vertigo with BVP (II), rapidly progressive BVP (III), slowly progressive BVP, and (IV) BVP with neurological deficits (such as ataxia, polyneuropathy).

The protocol for this study was approved by the institutional review board at our hospital. The research was under the World Medical Association Declaration of Helsinki.

### Audiometry

Audiometry was performed in a soundproofed booth (IAC mini 250), and the findings were reported in auditory thresholds of each frequency elicited (0.5, 1, 2, 3, 4, 6, and 8 kHz) and pure-tone averages (PTAs), which were computed by taking the average of the four frequencies (0.5, 1, 2, and 3 kHz).

### Video Head Impulse Test

We evaluated the dynamic function of the horizontal semicircular canals using the vHIT (GN Otometrics; Denmark). Fast, short, and unpredictable head impulses were performed in random horizontal directions while the subject was seated in front of the ground-fixed target and was instructed to maintain his/her vision continually fixed on the target during the test. Eye and head velocities were acquired with a sampling frequency of 250 Hz, and we calculated the hVOR gain from an average of 20 head impulses performed over a range of velocities from 100–250°/s.

The first pair of vertical canals was evaluated next. To do this, the patient's head was rotated 40° to the right to align it with the left-anterior/right-posterior plane. Patients were directed to continue staring at the same earth-fixed target as before. Brief, abrupt, forward, and backward head impulses were made to stimulate the left anterior semicircular canal and the right posterior semicircular canal, respectively. After 20 impulses in each direction, the second pair of vertical canals were evaluated. To do this, the patient's head was rotated 40° to the left while they continued staring at the same earth-fixed target as was used before to align the right-anterior/left-posterior plane. Forward and backward head impulses stimulated the right anterior semicircular canal and the left posterior semicircular canal, respectively.

The presence or absence of saccades, their latency, and amplitude were registered for each exploration. To assess the dispersion of the saccades' latency values, we used the PR score described by Rey Martinez et al. (12).

PR score is a quantitative variable that ranges between 0 and 100; when PR is close to zero, saccadic responses are said to be gathered, and when it is nearing 100 they are said to be scattered.

### Statistical Analyses

All data were stored and analyzed in an SPSS file version 25 (SPSS Inc.; Chicago, IL, USA). All tests were two-tailed, and *p* < 0.05 were considered significant. A χ^2^ test with Bonferroni's correction for multiple comparisons was performed to assess the differences in BVP detection. We conducted χ^2^ *post-hoc* tests based on adjusted standardized residuals (13).

Mann-Whitney *U*-test and Spearman's ρ were used to detect differences between measures within each series.

A multivariant ANOVA test was used to compare the auditory thresholds for every frequency and VOR gain between cohorts of patients.

## Results

In this study, 176 patients were initially included. The mean age of the patients was 61.70 ± 14.9 (range 14-90) at the time of the inclusion in the study.

Of our population, 56.8% were male and 43.2% female. The imbalance was perceived by 160 patients (96.2% of the population), while 76 (43.2%) experienced oscillopsia.

### Etiology

**Figure 1:**
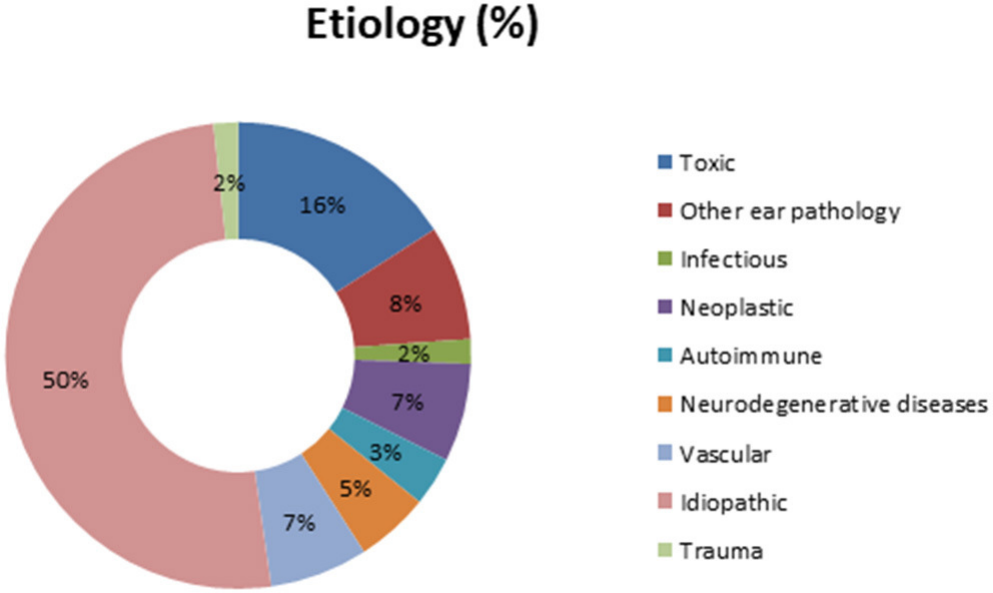
Distribution of etiologies in the study's population.

In our global population, the etiology for BVP was determined in 87 patients (50%). Meniere's disease, ototoxicity, and cerebellar infarction were the most prevalent diagnosis in those known etiologies. Figure 1 shows the complete distribution of etiologies in the study's population.

Of our population with Meniere's disease (MD), three cases had a synchronic MD, and seven cases developed bilateral metachronous MD.

Four patients of our MD population had unilateral disease with confirmed BVP, and all patients fulfilled the diagnostic criteria for definite MD. All those patients had a positive electrocochleography in both ears.

The group of neurodegenerative diseases consisted of nine patients with cerebellar atrophy, six of them with a confirmed CANVAS diagnosis.

Of our population, 117 patients (66.5%) had valid information regarding the familial history of chronic vertigo or disequilibrium. Fourteen patients had a first-degree relative, and five patients a second-degree relative. This makes 16.3% of patients with a prior family history, considering just those patients with relevant information of their familial background.

Among our patients, 13.6% had migraines in their medical history.

### General vHIT Parameters

Considering the total population, the mean gain was 0.60 ± 0.17 and 0.42 ± 0.18 for both better and worse horizontal semicircular canals. Mean values were 0.53 ± 0.27 and 0.43 ± 0.23 for better and worse posterior semicircular canals, respectively. Superior semicircular canals had 0.62 ± 0.27 and 0.49 ± 0.22 on their better and worse side, respectively.

A 41.6% of our population developed covert saccades and 87.6% had overt saccades on the worse side, while 32.6 and 66.4% of our patients had covert saccades and overt saccades, respectively, on the better side. There were statistically significant differences between both sides, either for covert saccades (Chi-square: 30.31; *p* = 0.001) or overt saccades (Chi-square: 15.06; *p* = 0.001).

The mean overt saccades latencies were 218.4 ± 65.1 and 226.3 ± 62.74 ms for the worse and better side, respectively. The mean covert saccades latencies were 112.32 ± 26.5 and 109.5 ± 28.26 ms for the worse and better side, respectively. We didn't find any statistically significant differences between both sides, either in overt or covert saccades latencies (*p* < 0.05).

The PR score had a mean value of 46.45 ± 26.89 on the better side and 50.38 ± 27.57 on the worse side, without statistically significant differences.

### Auditory Results

The PTA of the global population was 40.26 ± 29.17 and 32.96 ± 24.19 for the worse and better side, respectively. No correlation was found between the PTA and VOR gain values.

### Clinical Subtypes of BVP

Slowly progressive BVP without vertigo, followed by recurrent vertigo with BVP, was found in 24.4% (*n* = 43) and 21.0% (*n* = 37), respectively. In the former subtype, idiopathic BVP was most accounted for, while Meniere's disease was the most frequent diagnosis in the latter. In 17% of our population (*n* = 30), a rapidly progressive BVP was diagnosed, due in many cases to ototoxicity, while 14.9% of our cases (*n* = 26) developed BVP with neurological deficit.

**Figure 2:**
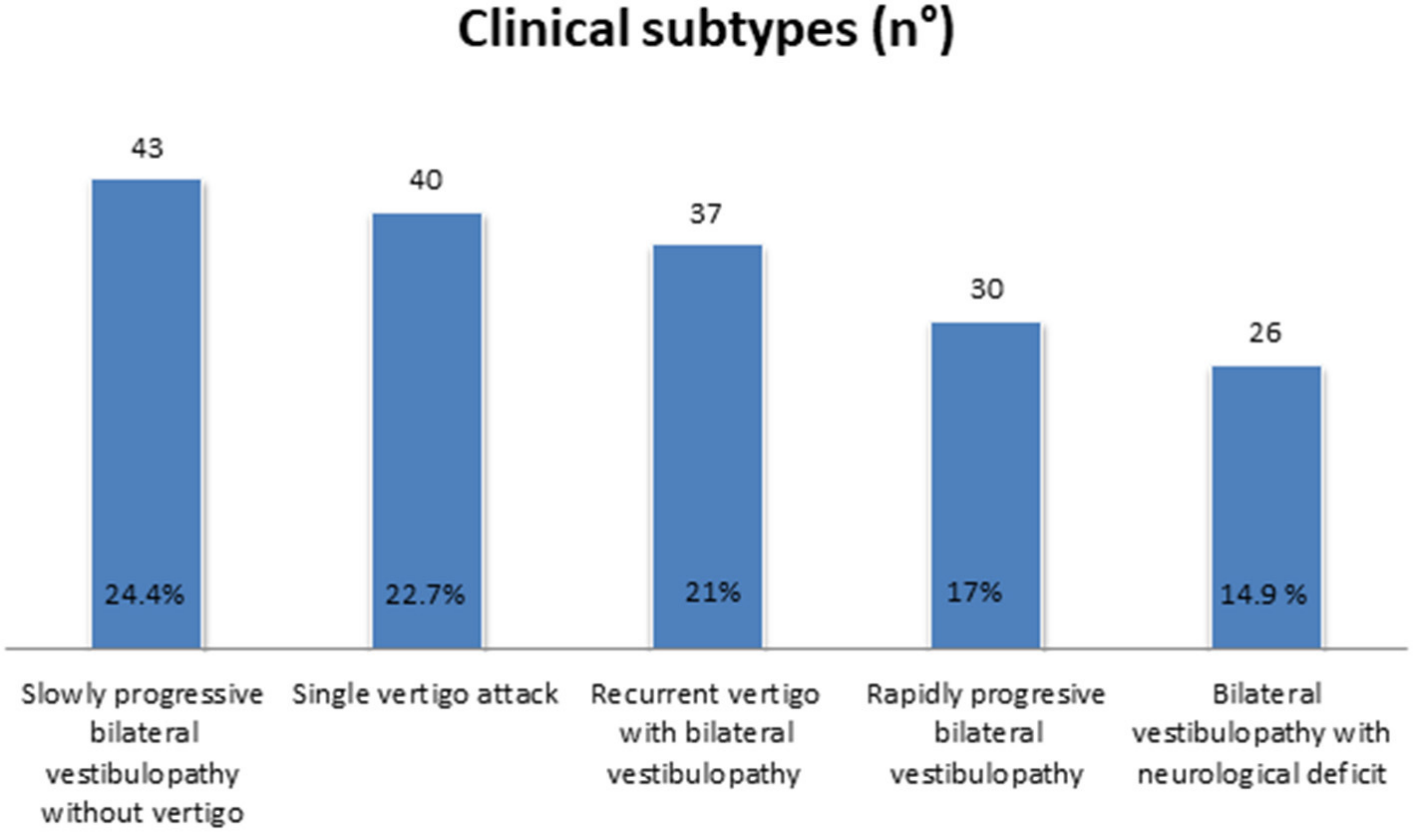
Clinical subtypes.

A significant percentage of our population could not be categorized in any of the former subtypes. Many of them were diagnosed with BVP after suffering a single vertigo episode (22.7%, *n* = 40), so a new subtype was added (Figure 2 and Table 1).

**Table 1:** Distribution of etiologies with respect to the clinical subtypes.

	**Slowly progressive bilateral vestibulopathy without vertigo**	**Single vertigo attack**	**Recurrent vertigo with BVH**	**Rapidly progresive BVH**	**BVH with neurological deficit (ataxia, polyneuropathy)**
Toxic	4	2	5	16	1
Other ear pathology	2	0	12	0	0
Infectious	1	0	0	1	1
Neoplastic	8	1	0	2	1
Autoimmune	2	0	1	2	1
Neurodegenerative diseases	2	0	0	0	7
Vascular	0	3	2	2	5
Idiopathic	24	33	17	5	10
Trauma	0	1	0	2	0
Total	43	40	37	30	26

**Figure 3:**
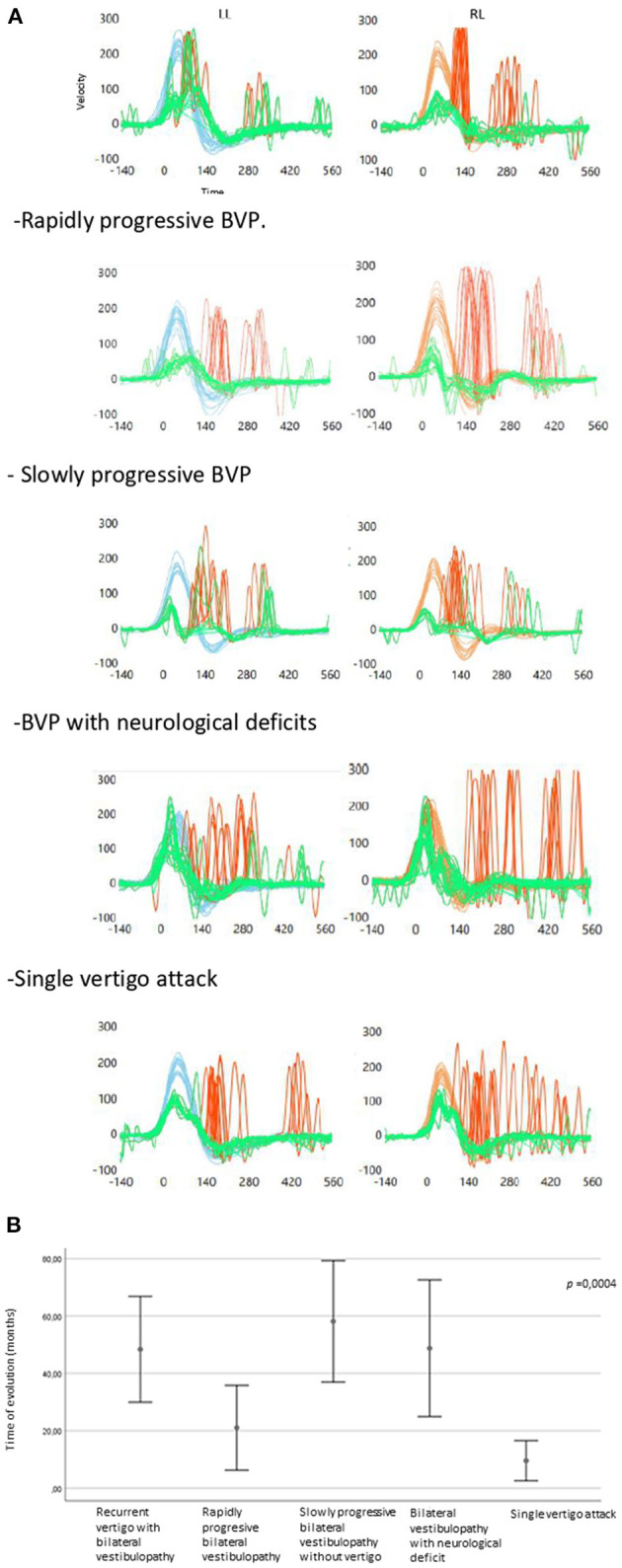
**(A)** CSH-VHIT examples according to clinical subtypes in the study's population (LL, Left lateral; RL, right lateral). Recurrent vertigo with BVP. **(B)** Error graph showing time of evolution regarding the clínica subtypes.

Regarding the vHIT parameters, the gain in the better horizontal semicircular canal was significantly higher in the single vertigo episode group compared to the other clinical subtypes (*p* < 0.05) (Figure 3A shows vHIT examples according to clinical subtypes).

The time of evolution differed significantly depending on the clinical subtypes of BVP (Figure 3B). The rapidly progressive BVP and the single vertigo attack subtypes had a shorter time of evolution and differed significantly from the rest of the groups.

### Barany Criteria

Eighty-nine patients (50.6%) fulfilled the diagnostic criteria for BVP proposed by Barany's society. The rest of our population fulfilled the diagnostic criteria for probable BVP.

The time of evolution was 41.67 ± 56.53 and 34.29 ± 50.09 months for those patients with and without Barany's criteria for BVP, respectively. We didn't find significant differences regarding the time of evolution between both groups.

**Figure 4:**
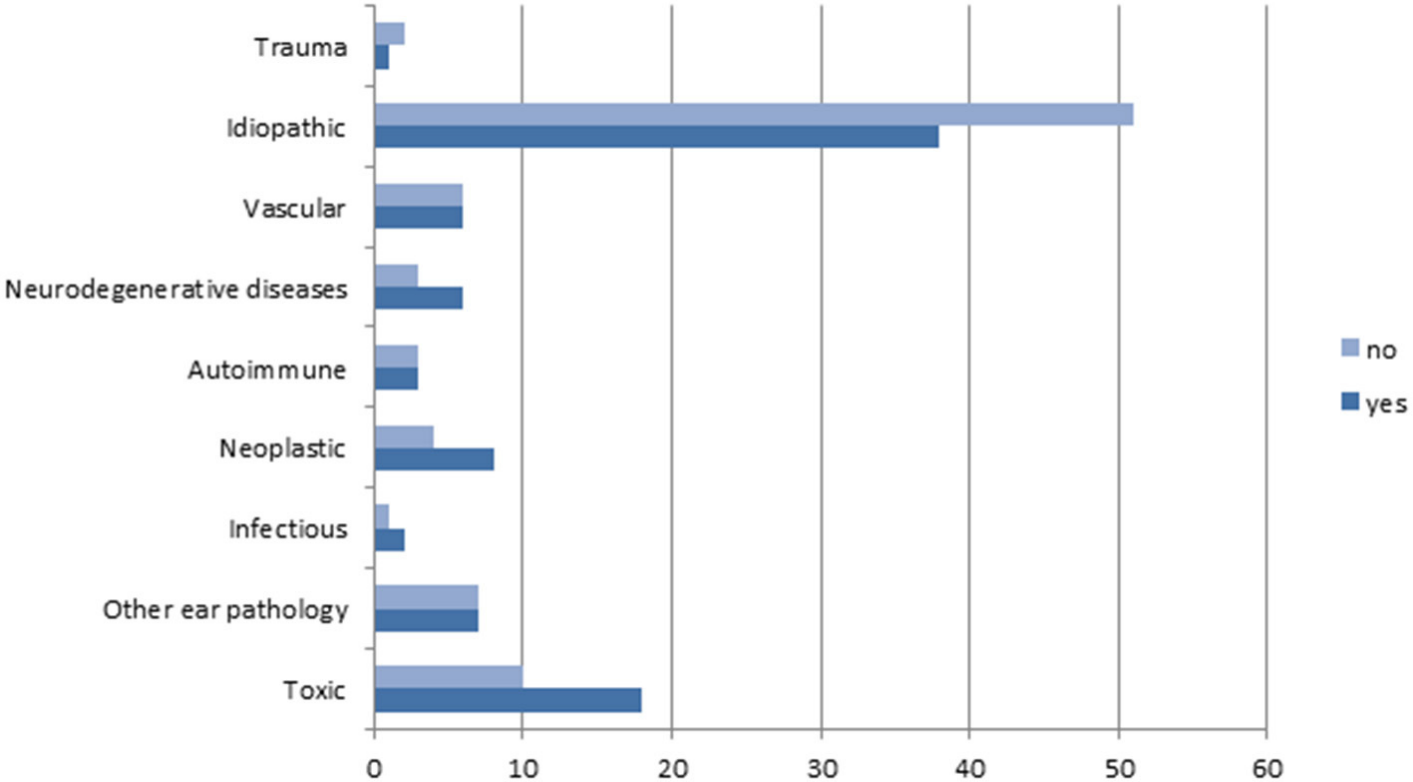
Distribution of the different etiologies according to the fulfillment of Barany's diagnostic criteria for BVH.

The global distribution of different diagnoses did not differ significantly between both groups (Chi-square: 7.53; *p* = 0.48), but idiopathic cases were significantly less prevalent (*p* = 0.024) in those patients who fulfilled Barany's diagnostic criteria for BVP. Figure 4 shows the distribution of the different diagnoses according to Barany's diagnostic criteria for BVP.

The imbalance was equally distributed in both groups of patients (Chi-square: 0.002; *p* = 0.962). Oscillopsia was significantly more prevalent in the group of patients that fulfilled Barany's criteria (Chi-square: 5.138; *p* = 0.023).

**Figure 5:**
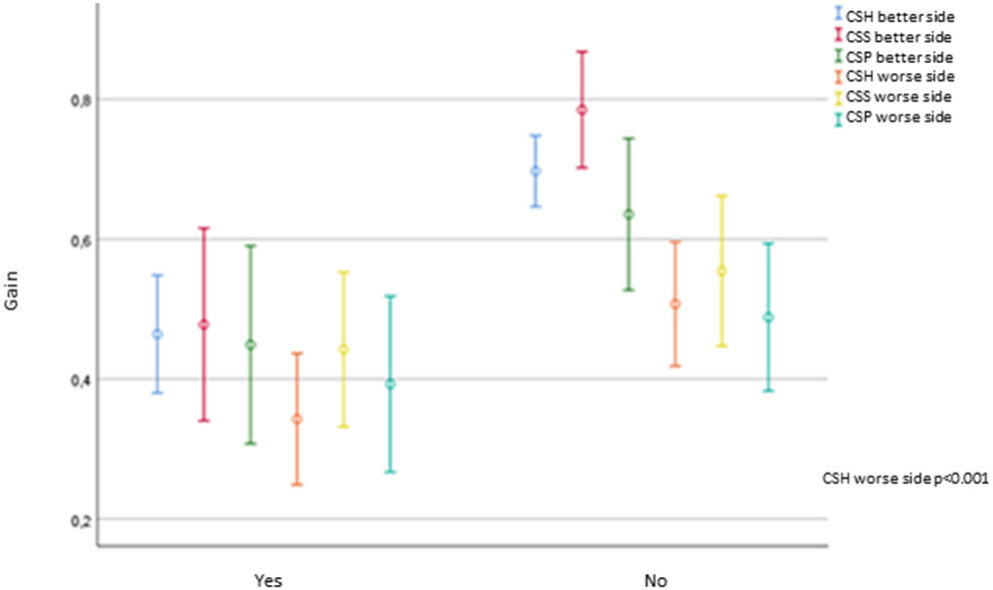
Error graph showing gain for each of canal on VHIT respect to Barany's criteria.

The gain of the worse horizontal semicircular canal was significantly lower in those patients with Barany's criteria for BVP compared to the probable BVP group (*p* < 0.001). No other vHIT parameters, such as the gain in the posterior and superior canal, PR score, the presence of saccades, or its latency, either in the better or the worse side, showed significant differences depending on Barany's criteria. Figure 5 shows the gain for each canal on vHIT concerning Barany's criteria.

Neither the PTA nor the auditory thresholds for every frequency elicited differed significantly depending on the fulfillment of Barany's criteria for BVP.

## Discussion

This study mainly focuses on the difficulties inherent in diagnosing a BVP in our population of 176 patients.

We had nearly half of our population with an unknown diagnosis, which makes idiopathic cases still be the barrier to demolish.

The most important variations in idiopathic cases among the different series are mainly explained by the population's heterogeneity, and the clinical setting in which the patients are seen. Being a neurological or an otorhinolaryngological department may influence the type of patients referred to and the medical follow-up performed. These variations may explain the different percentages of MD when comparing our population to other similar studies (4). Bilateral MD rarely presents with each ear simultaneously but rather sequentially with the second ear involvement occurring many years after the first (14). This fact is represented in our population, with three and seven cases of synchronic and metachronous MD, respectively.

Four patients of our MD population had definite unilateral disease based on Barany's guidelines. All those patients had a positive electrocochleography in both ears, suggesting the endolymphatic hydrops as possible but not confirmed, the underlying cause of their BVP.

The finding of bilateral vestibular involvement in a clinically suspected unilateral vestibulopathy is still one of the most challenging diagnoses and has significant pathophysiological implications. Recovery from vestibular disorders, regardless of the etiology, is partly mediated by this process of vestibular habituation (15), controlled by the central vestibular system.

A unilateral vestibular schwannoma with bilateral vestibular loss was found in 12 patients. Lucieer et al. (3) reported four similar patients, and this condition was also described by Pinna et al. (16). They described 12.7% of their population of 511 vestibular schwannomas, with contralateral hyporeflexia, and 6.5% with a total areflexia. Although some authors attribute contralateral hydrops as the possible explanation for this BVP, the possibility of a habituation process in slow-growing vestibular schwannomas should be noted.

Habituation of vestibular nystagmus has been observed among ballet dancers, pilots, and figure skaters. This fact could be linked to our three cases of symptomatic BVP developed after unilateral vestibular neuritis. Immediately following unilateral vestibular deafferentation, there is asymmetric nuclei activity where the intact side becomes initially increased, after which cerebellar inhibition depresses the activity from the vestibular nuclei bilaterally (17). Although cerebellar inhibition is supposed to be transitional, our finding raises the question of whether a unilateral vestibular loss can develop asymptomatic chronic BVP. In our three vestibular neuritis cases, all developed significant vestibular damage on one side with moderate affection of the contralateral one, thus developing BVP, which fulfilled Barany's diagnostic criteria.

The group of patients who fulfilled Barany's criteria for BVP had a trend toward a longer time of evolution, although those differences were non-significant. This together with the significant lower horizontal gains and the higher proportion of patients with oscillopsia could lead us to the conclusion that those patients will possibly develop a complete BVP syndrome, ulteriorly.

Regarding the vHIT parameters, our results are similar to those of other BVP populations with a significant decrease of the different semicircular canals gain and the consequent development of corrective saccades (1, 9).

The diagnostic criteria for BVP based on the consensus document of the Classification Committee of the Bárány Society specify that bilaterally reduced or absent angular VOR function documented by a bilaterally pathological horizontal angular VOR gain of <0.6, measured by vHIT or scleral search-coil technique, is mandatory for definite diagnosis (1). However, since the introduction of vHIT in clinical practice, the initial evaluation has been expanded to all six canals (1). Both new approaches (providing a more accurate degree of dysfunction and a specific pattern) present new insight to better understand the different degrees of disability and handicap known to occur in that population of patients (9).

Nevertheless, further analysis of caloric response or horizontal angular VOR gain upon sinusoidal stimulation on a rotatory chair might contribute to a better understanding of our BVP patients.

Although our findings elicited that after head impulses toward the worse side, the PR was higher than after those toward the side where a higher gain value was obtained, those results were non-significant. Our results are similar to those found by Batuecas et al. (9), but they found significant differences in the PR score between both sides. Those differences could be explained by the differences in etiologies or the time of evolution in each population.

All previously described clinical subtypes were identified in our population. Slowly progressive BVP without vertigo and recurrent vertigo with BVP groups were the most prevalent. Our findings are similar to those described by Lucieer et al. (3) either in the idiopathic or in the non-idiopathic cases. Nevertheless, in our population, a significant proportion of our patients developed BVP after a single episode of vertigo. Those patients had a significantly shorter time of evolution and a significantly higher horizontal semicircular canal gain in the better side compared to the rest of the groups except the slowly progressive BVP without vertigo.

Thirty-three out of 40 patients in the former group were idiopathic, although the clinical presentation was suggestive of an acute vestibular syndrome, such as vestibular neuritis. All those patients complained about unsteadiness or oscillopsia and had bilaterally diminution of the horizontal semicircular canal gain. Nevertheless, a significantly greater proportion of the latter group did not fulfill Barany's criteria for BVP. In our opinion, this fact together with the shorter time of evolution prompts us to consider the possibility of a transitory BVP.

Dix and Hood (18) described a significant percentage of their population of 274 patients with vestibular neuritis, with bilateral abolition of both caloric and rotational response. A mechanism of vestibular habituation suppression was suggested with a possible mechanism of central suppression.

About 20% of patients with a chronic stable unilateral vestibulopathy will continue to experience chronic postural imbalance and oscillopsia, the same symptoms that all patients with BVP, symptoms which constitute the so-called syndrome of chronic vestibular insufficiency (19).

A 66.5% of our population had valid information regarding the familial history of chronic vertigo or disequilibrium. We had a 16.3% of patients with a clinical presentation and family history of disequilibrium, suggestive of genetic predisposition. Unfortunately, no genetic investigations were performed, and most of those patients were idiopathic.

A clear limitation is the retrospective nature of the study. There is frequently an absence of data on potential confounding factors if the data was recorded in the past. It may be difficult to identify an appropriately exposed cohort and an appropriate comparison group. Further studies are needed and should address the issue to conduct prospective studies with appropriate comparison groups.

## Conclusions

BVP is a heterogeneous condition with a high proportion of idiopathic cases.

The distribution of the clinical subtypes and Barany's criteria are a useful clinical tool to differentiate groups of patients, and therefore predict its evolution.

The finding of bilateral vestibular involvement in a clinically suspected unilateral vestibulopathy should be considered in some patients.

## Data Availability Statement

The raw data supporting the conclusions of this article will be made available by the authors, without undue reservation.

## Ethics Statement

The studies involving human participants were reviewed and approved by institutional review board at our hospital. Written informed consent for participation was not required for this study in accordance with the national legislation and the institutional requirements.

## Author Contributions

FM-M and EM-S contributed to planning of the study, acquisition of data, statistical analyses, interpretation, and writing of the manuscript. AR and JE-S contributed to the planning of the study and acquisition of data. All authors contributed to the article and approved the submitted version.

## Conflict of Interest

The authors declare that the research was conducted in the absence of any commercial or financial relationships that could be construed as a potential conflict of interest.
